# The Precision Control of Autophagic Flux and Vesicle Dynamics—A Micropattern Approach

**DOI:** 10.3390/cells7080094

**Published:** 2018-08-03

**Authors:** André du Toit, Sholto De Wet, Jan-Hendrik S. Hofmeyr, Kristian K. Müller-Nedebock, Ben Loos

**Affiliations:** 1 Department of Physiological Sciences, Stellenbosch University, Stellenbosch 7602, South Africa; andredt@sun.ac.za (A.d.T.); 18455468@sun.ac.za (S.D.W.); 2 Department of Biochemistry, Stellenbosch University, Stellenbosch 7602, South Africa; jhsh@sun.ac.za; 3 Department of Physics, Stellenbosch University, Stellenbosch 7602, South Africa; kkmn@sun.ac.za

**Keywords:** autophagic flux, vesicle dynamics, autophagosome, autolysosome, lysosome, pool size, velocity, displacement, micro-pattern

## Abstract

Autophagy failure is implicated in age-related human disease. A decrease in the rate of protein degradation through the entire autophagy pathway, i.e., autophagic flux, has been associated with the onset of cellular proteotoxity and cell death. Although the precision control of autophagy as a pharmacological intervention has received major attention, mammalian model systems that enable a dissection of the relationship between autophagic flux and pathway intermediate pool sizes remain largely underexplored. Here, we make use of a micropattern-based fluorescence life cell imaging approach, allowing a high degree of experimental control and cellular geometry constraints. By assessing two autophagy modulators in a system that achieves a similarly raised autophagic flux, we measure their impact on the pathway intermediate pool size, autophagosome velocity, and motion. Our results reveal a differential effect of autophagic flux enhancement on pathway intermediate pool sizes, velocities, and directionality of autophagosome motion, suggesting distinct control over autophagy function. These findings may be of importance for better understanding the fine-tuning autophagic activity and protein degradation proficiency in different cell and tissue types of age-associated pathologies.

## 1. Introduction

The accumulation of toxic protein aggregates in neuronal cells is a major hallmark for brain aging. A tightly controlled molecular mechanism governing proteostasis is required to promote neuronal health, thereby impacting directly on cell viability and function [1,2]. Autophagy is a key protein degradation pathway that is crucial in not only degrading long-lived proteinaceous cargo, but also in finely tuning neuronal metabolism and activity with the cell’s metabolic demands [3,4]. Two major types of autophagic mechanisms participate in this quality control system. Macroautophagy (hereafter referred to as autophagy) primarily targets aggregated and misfolded proteins through autophagosomes for lysosomal degradation, while chaperone-mediated autophagy (CMA) operates through specifically targeted cytosolic proteins that are delivered to lysosomes via chaperones. Importantly, the rate of protein degradation through the entire autophagy pathway, i.e., autophagic flux, indicates the activity of this process [5] and has received major attention, since its decline contributes to cellular toxicity, degeneration, and aging.

It has long been recognized that mammalian tissues are characterized by a distinct, tissue-specific abundance of LC3 protein [6] which is also distinctively responsive to metabolic perturbations [7]. Moreover, recent advances in measuring autophagic activity, or autophagic flux, i.e., the rate of protein degradation through autophagy, have demonstrated that the basal rate of protein degradation under control conditions differs substantially between tissue types [8], allowing to rank organs and tissues according to their autophagic activity. In this context, it is of particular importance to note that some tissues are characterized by a particularly high autophagic flux, indicating a very efficient protein cargo clearing system [9], which declines in ageing and degeneration [10]. Autophagy dysfunction leads to protein aggregation, which enhances toxicity of the intracellular microenvironment, including mitochondrial quality control, further contributing to cellular pathology. Also, cancer cells are characterized by a heightened basal autophagic activity and by a change in their autophagy proficiency [11]. It is therefore not surprising that the pharmacological modulation of autophagy has attracted considerable attention as a potential therapeutic target in pathologies associated with autophagy dysfunction [12] and a multitude of drug libraries is being screened for most effective autophagy inducing or inhibiting properties [13]. A primary focus in the context of neurodegeneration and aging is thereby, to better identify the autophagy defect, to offset autophagy dysfunction and to enhance protein clearance by inducing autophagy [14], in order to preserve neuronal cell viability. Similarly, in many cancer types, clinical trials are on the way with the aim to either robustly or sequentially [15] induce the autophagic system to inherently lethal or sensitizing levels, or to inhibit the pathway, so as to sensitize to cell death induction in combination with a chemotherapeutic agent [16]. 

However, the level of the most desirable level of autophagic activity, related to the given pathology, i.e., the most suitably level of protein clearance, remains thus far largely unclear. This is, at least in part, due to the challenge of measuring autophagic activity precisely and controlling pathway intermediate steady-state pool sizes [17]. Hence, the precision control of autophagy, to better tune the autophagic machinery and its activity, is highly desirable, however, remains poorly assessed. How far could autophagy be functionally exploited, without leading to vesicular crowding and organelle traffic failure? Critical in this context is the role of the microtubule network, its carrying capacity, to deliver vesicular cargo such as autophagosomes, ATP dependently to the perinuclear region, where fusion with lysosomes is facilitated. Although such autophagosome vesicle dynamics have been described in the past [18], the relationship between autophagic flux, pathway intermediates and their pool sizes and well velocities remain largely unclear. In particular, it is not known whether enhanced autophagic flux will lead to an increase in autophagosome vesicle velocity or displacement, thereby enhancing autophagy function, or whether autophagy vesicle dynamics are equally dependent on mTOR dependent or independent autophagy induction. Finally, although it has recently been revealed that hyperosmotic and mechanical stress exerts a degree of control on autophagic activity [19] it remains unknown to what extent geometric constraints, such as cellular size and shape, may affect the response to autophagy modulation. The rational of this study was, therefore, to utilize a basic mammalian model system, allowing to assess two autophagy inducing drugs and to dissect the relationship between autophagic flux and vesicle dynamics. To enhance experimental control and to include geometry constraints, allowing to assess the contribution of tubulin organisation in this context, micropatterning was additionally utilized. 

The above findings strongly demand the integration of an experimental approach that allows to describe autophagic flux and pathway intermediates in a highly controlled cellular environment, so as to better understand vesicle dynamics and autophagosome/lysosome fusion behaviour, as an indication of functional protein degradation through autophagy. Recent advances in autophagic flux assessment and control of pathway intermediates [17,20,21] and enhanced approaches of micropatterning [22] provide a new level of control over autophagic activity as well as geometry constraints, allowing to dissect pathway intermediates, their velocities and tubulin structure with high precision. In this context, mouse embryonic fibroblasts, in particular, have been utilized as suitable mammalian cell model systems [8,21].

Therefore, the aims of this study were to firstly establish conditions of precision-controlled autophagic flux, assessing two known autophagy modulators, spermidine and rapamycin, using a concentration that achieves similarly raised autophagosome flux. Taking advantage of a precisely determined autophagosome flux, we next assessed the corresponding intracellular pathway intermediate pool sizes, i.e., autophagosome, autolysosomes and lysosomes. Secondly, we aimed to determine whether manipulation of the autophagic pathway with known fluxes leads to a change in autophagosome velocity, displacement and directionality, as an indication for successful cargo delivery and functional autophagy. Finally, by implementing a micropatterning approach, in order to maximize control over cellular morphology, we aimed to resolve the role of physical constraints, cellular shape and the tubulin network geometries on autophagy vesicle dynamics and hence autophagy function. Our results reveal a surprising differential effect of autophagic flux enhancement on autophagosome velocity, position and motion as well as displacement when comparing mTOR dependent and mTOR independent pathway activation, with a distinct impact of cellular geometry, blunting autophagosome velocity and redirecting autophagosome position. These data highlight distinct cellular responses of autophagy enhancing drugs and reveal an additional level of control through cellular morphology and shape, requiring careful autophagic flux titration in a cell and tissue specific manner when screening autophagy modulating drugs for clinical translation. The results further suggest that cellular shape and tubulin network geometries, as well as a defined tissue microenvironment, may impact autophagy function, by distinctly affecting vesicle motion, fusion and hence the cell’s protein degradation proficiency.

## 2. Materials and Methods

### 2.1. Cell Culture 

Mouse Embryonic Fibroblast (MEF) cells that stably express GFP light chain 3 (LC3) protein (a kind gift from Prof. Noboru Mizushima, Tokyo University, Japan) were cultured in Dulbecco’s modified Eagle’s medium (DMEM) (Life Technologies, 41965-039) supplemented with 10% fetal bovine serum (Biochrom, Berlin, Germany, S-0615), 1% Penicillin-Streptomycin (ThermoFisher, Johannesburg, South Africa, 15140148); 100 U/mL of penicillin and 100 μg/mL of streptomycin, and maintained in a humidified atmosphere in the presence of 5% CO_2_ at 37 °C. The sub-culturing of MEF cells involved trypsinisation (Life Technologies, 25200-072) to dissociate adherent cells from the flask. Following trypsinisation, cells were collected in a 50 mL falcon tube (Biocom Biotech, Centurion, South Africa, 50050) and supplemented DMEM was added in a 2:1 ratio. Cells were centrifuged (Merck Millipore, Johannesburg, South Africa, Eppendorf Centrifuge 5804 R) at 1500 rpm for three minutes at room temperature. The supernatant was discarded, and cells re-suspended in DMEM. Cells were seeded in either culturing flasks (Porvair, WhiteSci, Brackenfell, South Africa, 500030), in an 8-chamber cover slip-based dish (Nunc Lab-Tek, Thermo Scientific, Johannesburg, South Africa, 155411PK) or a micropatterned slide for experimental purposes.

### 2.2. Fabrication of Micropatterned Slides

Fibronectin disc shapes were micropatterned on glass using deep UV as described by Carpi et al. [23] with some modifications. The glass coverslip was washed with 70% ethanol, dried using a dry-oven and then silanizated by deep UV (185 nm) illumination for 10 min using a UVO cleaner (Jelight, model 18). Thereafter the glass slide was passivated by placing it on top of a drop of PLL-g-PEG solution (0.1 mg/mL PLL(20)-g[3.5]-PEG(2) (Surface Solutions, Zurich, Switzerland), 10 mM Hepes, pH 7.4.) on a sheet of parafilm with the activated face in contact with the PLL-g-PEG. The coverslip was incubated with the PLL-g-PEG solution for one hour at room temperature. The coverslip was then washed for 2 min in PBS and rinsed for 2 min in millipore water. A quartz photomask with the edged patterns was used to transfer the patterns onto the PLL-g-PEG layer. The mask was manufactured by Delta Mask, Toppan, Netherlands, using a GDSII layout file that was created using the Python platform and gdsCAD 0.4.5 package containing the pattern designs; i.e., circle patterns with a diameter 50 μm. The photomask was washed with 70% ethanol and dried using absorption paper. The non-chrome side of the photomask was silanated by placing it with the chrome side facing down in the UVO cleaner and illuminating it for 10 min. The photomask was left to cool and then 10 μL millipore water was placed onto the non-chrome surface of the mask. The coverslip with the activated face was then placed on the drop of millipore water in contact with the photomask. Excess water was removed using absorbent paper. The photomask was placed with its chrome side towards the lamp in the UVO cleaner and illuminated for 10 min. Next, the mask was left to cool down and then submerged in millipore water with the chrome side facing down (the coverslip facing upwards) until the coverslip spontaneously started to lift off. The coverslip was carefully removed to avoid scratching the mask and placed on a 70 μL drop of fibronectin solution (25 μg/mL fibronectin (Sigma, Johannesburg, South Africa, F1141), 100 mM NaHCO3, pH 8.5.) on parafilm with the illuminated surface in contact with the solution and incubated for 2 h at room temperature. Thereafter the coverslip was washed for 2 min in millipore water and then dried overnight at 4 degrees and glued to the microscope slide fabricated from acetal. Cells were harvested by trypsinisation and 1000 cells were added to each well and placed in the incubator for 1–2 h to allow them to adhere. Thereafter, unattached cells were washed off using culture media.

### 2.3. Treatment Conditions

A cocktail of fluorescent probes was prepared containing 75 nM LysoTracker Blue (ThermoFisher, Johannesburg, South Africa, L7525) and 100 nM SiR-tubulin (SpiroChrome, Stein am Rhein, Switzerland, CY-SC002) in culture media. Media was refreshed with culture media containing the dyes and the respective drugs; 10 nM Bafilomcyin (LKT labs, B0026), 200 nM Rapamycin (Sigma, R8781) and 20 nM Spermidine (Sigma, S2626). For autophagic flux analysis, saturating concentrations of Bafilomycin (400 nM) were utilized, and autophagosome pool size quantified as previously described [21]. Cells were treated for 8 h and then either imaged or harvested for western blot analysis. 

### 2.4. Live Cell Imaging

Slides containing MEF cells were placed in a humidified chamber at 37 °C supplemented with 5% CO_2_ that encases the stage of the Carl Zeiss (Oberkochen, Germany) LSM780 P.S.1 microscope. The raw image series was acquired using a high NA objective (Olympus Plan APO N 60×/1.42 Oil/0.17/FN26.5) with 20 image acquisition cycles in 15 s intervals. An Argon multiline laser 25 mW at 488 nm and Diode 405 nm CW/PS and 633 nm laser were used as light source in combination with a GaAsP detector. Laser power and master gain were chosen for 405 nm (LysoTracker blue), 488 nm (GFP) and 633 nm (SiR Tubulin) to ensure an optimal signal/noise ratio with minimal saturation using MBS: MBS 488/561/633 and MBS_InVis: MBS 405 beam splitters. Track filters were set as follows; LysoTracker: 410–471 nm, GFP: 490–597 and SiR Tubublin: 638–759 nm.

### 2.5. Image Analysis 

Images were processed using ZEN 2011 imaging software (Carl Zeiss, Oberkochen, Germany) and exported to an automated puncta counting and tracking software, ImageJ with TrackMate plug-in [24], for analysis.

We analysed the positions and motion of tracked, fluorescently labelled autophagosome puncta for two cells, one circularly patterned (MP) and the second not patterned (NP). For the circularly patterned cell 323 individual puncta were tracked over 7 median number of consecutive frames, whereas the NP-dataset had 340 puncta over 13 median number of frames. 

A potential source of differing behaviours in these two cases is the underlying arrangement of microtubules, as is known from stiff confined filaments. In a disk-like structure, a natural point of reference is the centre. We introduce quantities to discriminate whether the motions of puncta are mainly radial (towards or away from the centre) or perpendicular to the radial vector (i.e., parallel to the boundaries). This measure of order depends on puncta positions and velocities

(1) $$Q=\frac{1}{N\left(S\right)}\underset{n\;\in \;S}{\sum }(1-2\;{({\hat{r}}_{n}\cdot {\hat{v}}_{n})}^{2})$$

where the sum is over a set of N(S) puncta S, ${\hat{r}}_{n}$ represents the unit vector from a fixed reference point to the punctum n, and ${\hat{v}}_{i}$ the velocity vector of the punctum (calculated by comparing positions of the same punctum at different frames/time-steps). For particles which move on average isotropically Q=0, whereas particles moving mainly towards or away from the central reference position would show a Q<0. Q is positive for motion that is perpendicular to the radial vector. The maximum of Q is 1 and its minimum −1.

We also determined the radial current and the average radial component of the velocity per punctum via

(2) $$I=-\underset{n\;\in \;S}{\sum }{\hat{r}}_{n}\cdot {\vec{v}}_{n},\;i=-\frac{1}{N\left(S\right)}\underset{n\;\in \;S}{\sum }{\hat{r}}_{n}\cdot {\vec{v}}_{n}.$$

These quantities are positive for inward net flow.

For the circularly patterned cell (MP) 323 individual puncta were tracked over 7 median number of consecutive frames, whereas the NP-dataset had 340 puncta over 13 median number of frames. The central reference point (in each case) was determined by calculating the centre of mass of all individual particles at the initial time measured. The farthest distance from this centre in MP was 24.1 units and in NP 57.5 units.

### 2.6. Western Blot Analysis

For protein extraction, culture medium was removed and cells were rinsed twice with cold PBS. 300 μL of RIPA buffer (50 mM Tris-HCl, 1% NP-40, 0.25% Na-deoxycholate, 150 mM NaCl, 1 mM EDTA, 1 mM PMSF, 1 mM Na_3_VO_4_, 1 mM NaF, 1 μg/mL leupeptin, 1 μg/mL aprotonin, 1 μg/mL benzamidine, 10 μg/mL pepstatin, pH 7.4) were added per flask and cells scraped off using a cell scraper (Porvair, WhiteSci, Brackenfell, South Africa, 500034). Lysates were collected and sonicated on ice using a Misonix (Fisher Scientific, Loughborough, UK, S-4000), and centrifuged at 8000 rpm at 4 °C for 10 min (Labnet International, Edison, NJ, USA, Spectrafuge 16M). Supernatant was collected and stored at −80 °C. A Bradford protein assay was used to determine the protein concentration of the lysates. For western blotting 40 μg of protein was mixed with Laemmli buffer (6.5 mM Tris-HCl, 2% sodium dodecyl sulphate, 5% mercaptoethanol, 10% glycerol, 0.01% Bromophenol blue, pH 6.8) in a 2:1 ratio and boiled at 95 °C for 5 min. Proteins were separated on a 12% TGX™ FastCast™ polyacrylamide gel (Bio-rad, Johannesburg, South Africa, 1610174) and transferred onto a Transfer Pack PVDF membrane (Bio-rad, 170-84156) using Trans-Blot Turbo (Bio-rad, 170-4155). Membranes were blocked with 5% milk in TBS-T (137 mM NaCl, 20 mM Tris, 0.1% Tween-20, pH 7.6) for 1 h. Thereafter membranes were washed with TBST three times for 5 min and incubated with primary antibodies overnight at 4 °C. The primary antibodies used were rabbit anti-LC3 (Sigma, L-8918 and anti-Sequestosome1/p62 (Abcam, Pretoria, South Africa, ab-56416). Membranes were rinsed with TBST three times for 5 min and then incubated with peroxidase-linked anti-rabbit IgG (Sigma, A-0545) for one hour at room temperature and visualized using Clarity Western ECL substrate and the ChemiDoc System (Bio-rad).

### 2.7. Statistical Analysis

Statistical analysis was performed using Statistica (13.0) using a one-way ANOVA and Bonferoni correction as well as LSD Post-Hoc tests where *p* < 0.05 was considered significant. 

## 3. Results

### 3.1. Precision Control of Autophagic Flux—Achieving Similar Autophagic Activity Via mTOR Dependent and Independent Pathway Induction 

Single cell analysis enables to precisely dissect the relationship between autophagic flux and autophagy pathway intermediates vesicle dynamics. In order to assess the role of increased autophagic flux on vesicle dynamics, their motion and directionality, an experimental condition was implemented that achieved similar autophagic activity using two different autophagy inducers, using non-patterned and patterned cells. 

**Figure 1:**
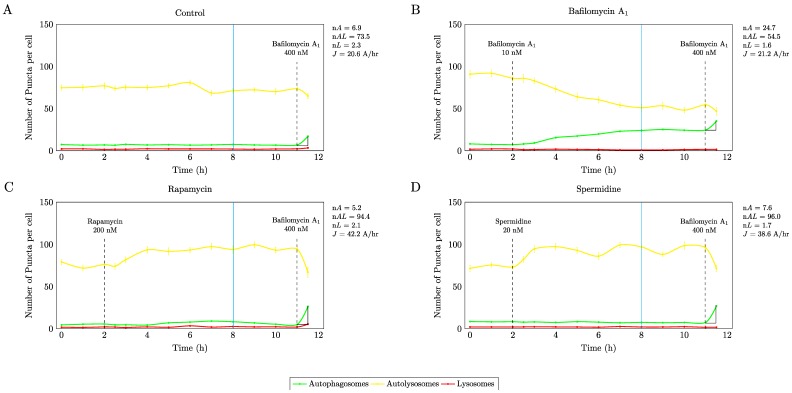
In order to assess autophagosome flux, the pathway intermediates were plotted in time. Flux response analysis using autophagy modulating drugs reveals a similarly enhanced autophagosome flux using 200 nM rapamycin (**C**) and 20 nM spermidine (**D**) compared to control conditions (**A**), with a new steady state flux established 6 h into the treatment time (blue vertical line). Note, low concentrations of bafilomycin lead to a change in pathway intermediate pool size (**B**), without impacting basal autophagosome flux. A total of 20 cells have been analysed.

Autophagosome flux was determined using the non-patterned cells, which were treated after 2 hrs with the respective autophagy modulating drugs, followed by complete inhibition of autophagosome/lysosome fusion with saturating concentrations of bafilomycin 9 h later (Figure 1). These results demonstrate that firstly with all autophagy modulating drugs, a new steady state was established after 6 h treatment intervention, which was indicated by no net change in the autophagy pathway intermediates. Secondly, both autophagy inducing drugs, i.e., 200 nM rapamycin and 20 nM spermidine resulted in an equal response in magnitude, resulting in a similarly enhanced autophagic flux (∆*J* ≈ 100%) raising from 20.6 A/h at basal levels to 42.2 A/h and 38.6 A/h respectively under induced conditions. 

Moreover, our results show that both rapamycin and spermidine raised autolysosomal pool size *nAL* from a basal pool of 73.5 ± 0.62 autolysosomes/cell to 94.4 ± 0.4 and 96 ± 1.23 autolysosomes/cell, suggesting that increased autophagosome flux manisfests largely in a change of the autolysosomal pool. Of note, only partial inhibition with 10 nM bafilomycin led to a distinct increase in the autophagosomal pool size (Figure 1B), however, with no change in autophagosome flux compared to non-treated control cells. These data indicate, that the same increased autophagosome flux can be achieved using mTOR dependent and independent pathway targeting strategies. These conditions were hence used for subsequent experiments. 

### 3.2. Precision Control of Cell Size and Tubulin Network—Assessment of Pool Sizes and Vesicle Trafficking

**Figure 2:**
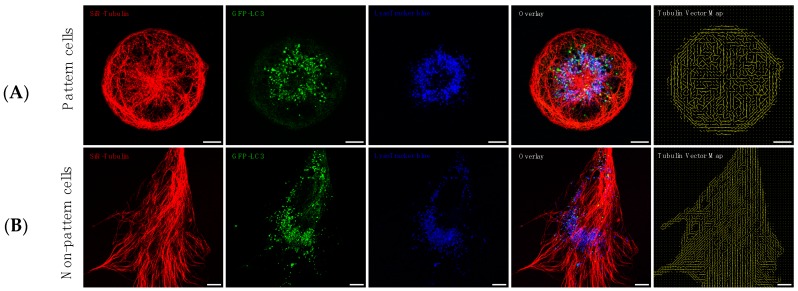
In order to enhance experimental control and to include geometry constraints, allowing to assess the contribution of tubulin organisation to autophagy pool size and vesicle dynamics, micropatterning was utilized. Micropatterned cells (**A**) display primarily perinuclear and branched organisation of autophagy pathway intermediates and a highly ordered tubulin network, while non-patterned cells (**B**) are characterized by a wide and asymmetric cytoplasmic distribution profile of autophagy pathway intermediates.

We evaluated the role of increased autophagosome flux on pathway intermediates, autophagosome velocity and displacement, using non-patterned and patterned cells (Figure 2) in order to control cellular geometry. Micropatterning resulted in a precise cellular size, a highly ordered cellular architecture with a distinctly organized tubulin network characterised by a high degree of symmetry (Figure 2A) and a primarily perinuclear distribution of autophagosomes and lysosomes. Non-patterned cells, on the other hand, displayed a completely asymmetrical tubulin network architecture with a wider distribution spectrum of autophagosomes and lysosomes throughout the cytoplasm and cellular processes (Figure 2B).

**Figure 3:**
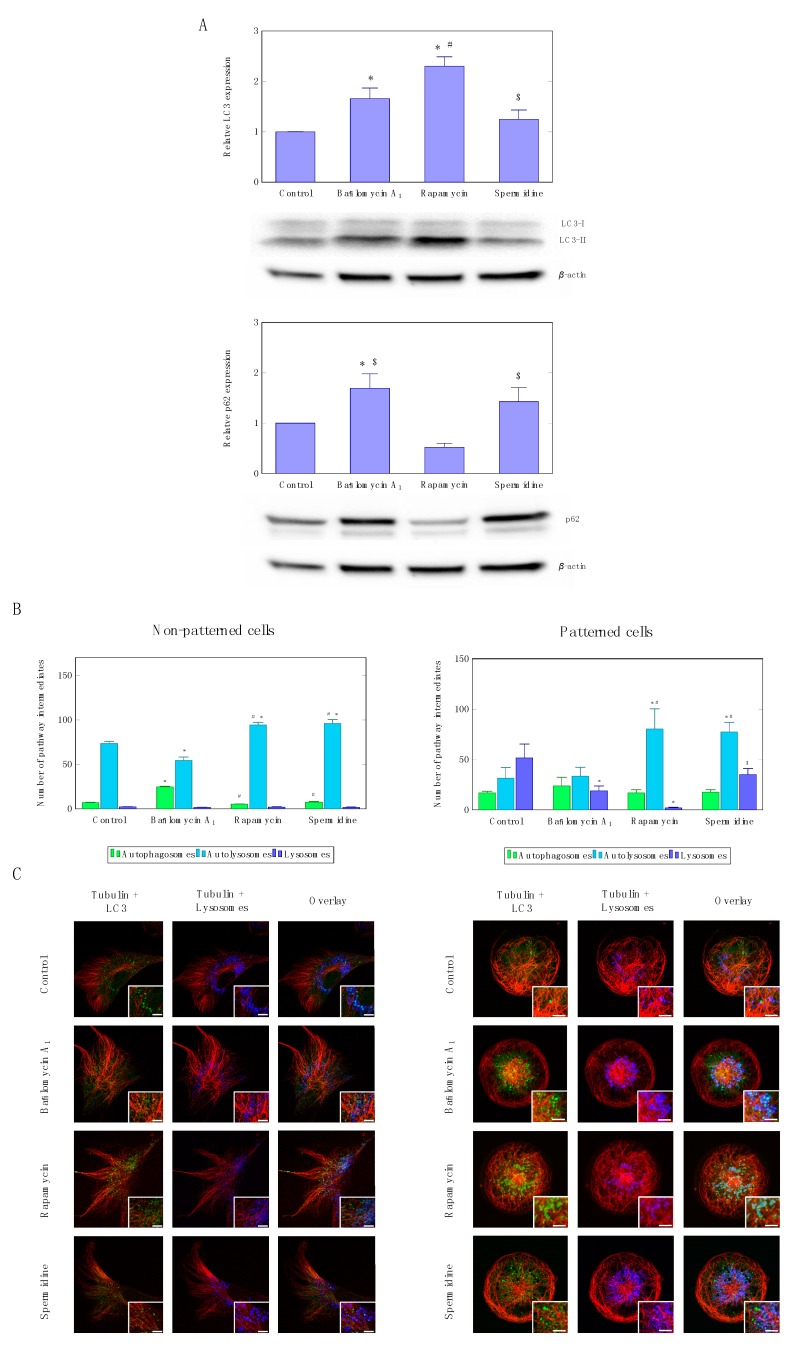
LC3-I/II as well as p62 protein expression levels (**A**) upon autophagy induction, using rapamycin or spermidine, as well as partial inhibition, using non-saturating concentrations of bafilomycin. A representative blot is shown. A minimum of three independent experiments (*n* = 3) were performed. Autophagy pathway intermediate pool size quantification (**B**) and representative micrographs (**C**) of non-patterned and patterned cells.

Although flux enhancement was similar when using rapamycin and spermidine, western blot analysis following autophagy induction revealed at steady state a significant increase in LC3-II protein levels only in the rapamycin treatment group compared to control conditions (Figure 3A)., Also sequestome/p62 protein levels decreased only upon rapamycin treatment, and increased upon spermidine treatment as well as partial inhibition using low concentrations of bafilomycin (Figure 3A).

These data are supported by the quantitative assessment of pathway intermediates (Figure 3B) as well as the fluorescence micrographs (Figure 3C and Figure S1), depicting a similar scenario, where similar fluxes not necessarily reflect in equal steady state protein levels of LC3-II and p62. They may also reflect that the turnover rate of autolysosomes may affect the protein levels of LC3-II, although the pool sizes of autophagosomes, autolysosomes and lysosomes were similar in both autophagy inducing conditions (Figure 3B). Induction of autophagy led to a significant increase in autolysosomes when using rapamycin and spermidine in non-patterned as well as patterned cells (Figure 3B). Micropatterned cells displayed an enhanced resolution in dissecting the lysosome pool size, which decreased upon rapamycin treatment, but not so in the spermidine treated cells. Colocalisation analysis of microtubule signal and autophagosomes indicates an equal network occupancy, with no differences under non-patterned or treatment conditions (Figure S2), however, rapamycin treatment appears to increase colocalisation area and occupancy in patterned cells. Moreover, overall autophagosome size appears to be larger in the rapamycin-treated cells, compared to autophagosomes observed in spermidine exposed cells (Figure S1). 

### 3.3. Precision Control of Cell Size and Tubulin Network—Assessment of Autophagosome Trafficking

**Figure 4:**
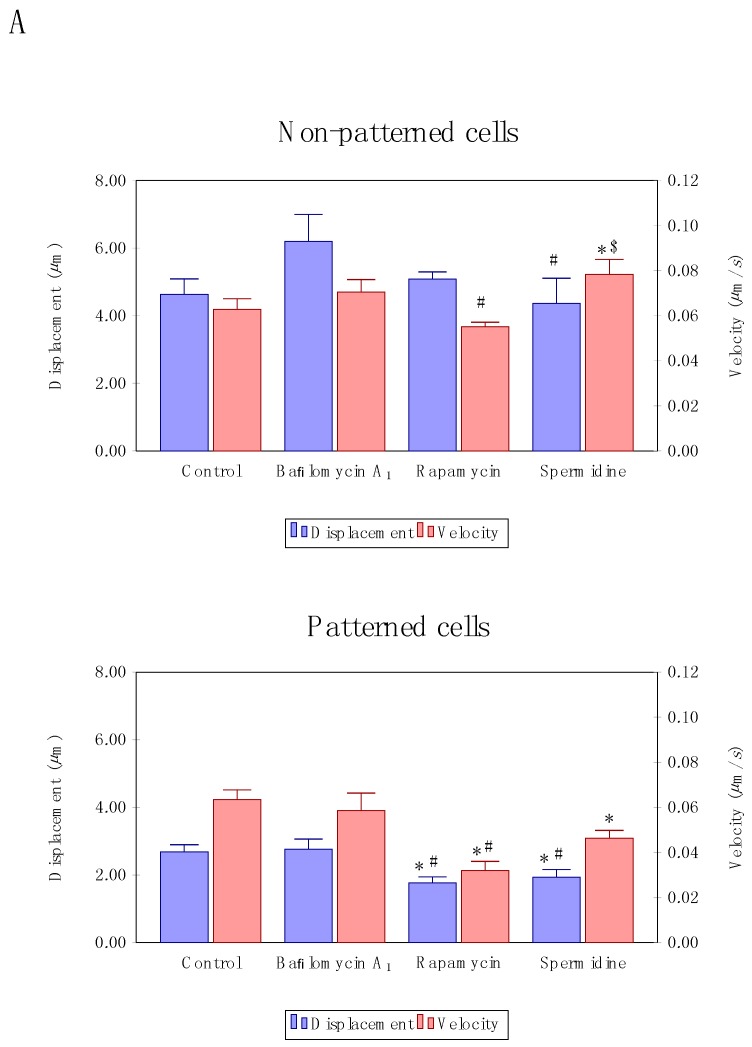

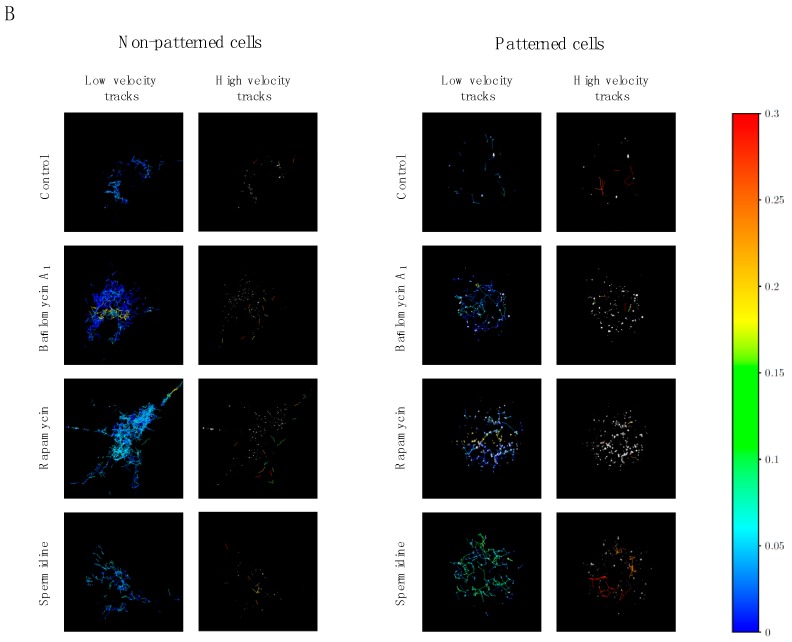
Autophagosome velocity and displacement analysis (**A**) in patterned and non-patterned cells upon autophagy induction, using rapamycin or spermidine, as well as partial inhibition, using non-saturating concentrations of bafilomycin. Representative micrographs of low and high velocity tracks (**B**) are indicated. A minimum of three independent experiments (*n* = 3) were performed.

To our surprise, velocity and displacement analysis revealed distinct differences between treatment modalities as well as non-patterned and patterned cells. Non-patterned cells were characterized by a significantly increased autophagosome mean velocity upon spermidine treatment (Figure 4A) compared to control cells as well as cells treated with rapamycin. In contrast, patterned cells revealed a significantly decreased mean velocity as well as mean displacement upon flux induction (Figure 4A) compared to control conditions. Moreover, partial inhibition and residual autophagic flux did not lead to a change in vesicle dynamics, remaining higher than those observed in the groups with enhanced autophagic flux (Figure 4A). When assessing vesicle tracks according to highest and lowest velocity, no distinct intracellular regional preference could be observed in both non-patterned and patterned cells (Figure 4B).

These data suggest that increased autophagic flux not necessarily increases autophagosome velocity or movement characteristics, in contrast, it may even decrease in conditions of geometry constraints, even though concentration and duration of autophagy modulation remains similar. Whether such response implicates functional protein degradation capacity, remains to be elucidated. When assessing high versus low velocity tracks in patterned and non-patterned cells, no preferred region of enhanced or decreased velocity is apparent (Figure 4B), suggesting that tubulin network organisation relative to the perinuclear region and cellular size is unlikely implicated in governing regional autophagosome velocity and displacement.

### 3.4. Directionality of Autophagosome Motion 

**Figure 5:**
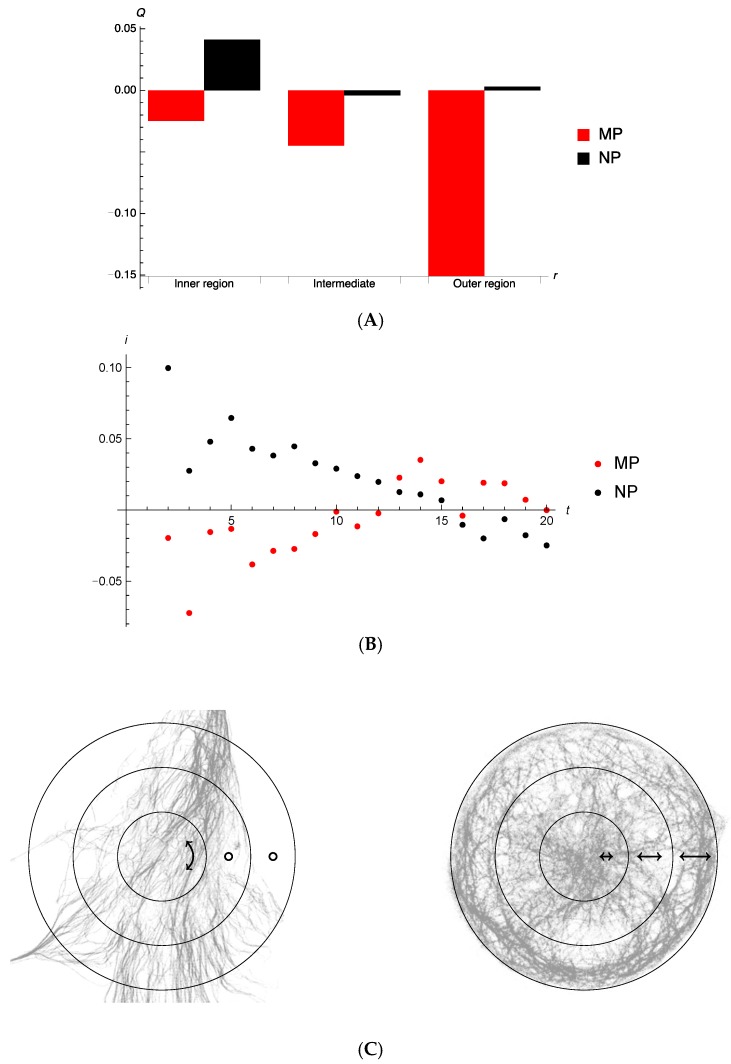
(**A**) Order measure Q as a function of radial distance for different segments of radius. No trend is discernable for the non-patterned cells (NP), but a clear radial motion, albeit rather small, for micropatterned cells (MP). In the micropatterned cells, autophagosome motion seems to be towards the periphery of the cell. (**B**) Average radial component of velocity per autophagosome punctum, as measured over 2 to 20 time-steps. For micropatterned cells, labelled particles initially move slightly towards the edge of the cell. For non-patterned cells, labelled particles are moving towards the centre. (**C**) Motion is mainly directed along the double-headed arrow line. A minimum of three independent experiments (*n* = 3) were performed.

In order to assess the directionality of vesicle movement in non-patterned and patterned cells, vector analysis was performed. For micropatterned cell, calculating the order measure for all time steps of all puncta, we determine Q=−0.04 (with a large standard deviation 0.7). We see, in Figure 5A, that average motion of autophagosomes in micropatterned cells has a weak trend to being radial which becomes stronger towards the periphery. No such trends are noticeable for the non-patterned cells. For this calculation the velocity of an autophagosome punctum was taken as the difference in displacement vector between two consecutive time measurements.

Considering that between single consecutive tracking events (frames or time-steps) autophagosomes typically only moved very small distances, we explored calculating velocity-dependent properties of puncta using a larger time-step separation, enabling longer distances for average autophagic punctum velocity determination, and less dependent on the system resolution. Of course, fewer autophagosomes are tracked over a longer period of time, which lowers the number of puncta that can be taken into account. The average radial velocity per autophagosome punctum tracked is shown for the non-patterned and micropatterned cells in Figure 5B. Of note, for the initial time frames autophagosomes in micropatterned and non-patterned cells seem to be moving in opposite directions. Any distinctive inward or outward motion for puncta that can be tracked beyond 10 frames is not discernible.

The quantity $Q_{\tau }$ as a function τ is the number of frames from the initial frame over which the particle is tracked. We have separated the MP sample into puncta captured within central, intermediate, and peripheral regions. The data indicate that those puncta moving in the periphery, and that are tracked for longer than approximately 10 time-steps, show rather distinctive behaviour of moving parallel to the confining boundary (indicated by a positive $Q_{\tau }$). This is not observed in the regions closer to the centre, nor do we observe positive order when particles are tracked over short times. It is to be noted that for small times τ, there are far greater numbers of puncta to be tracked. (please see Supplementary Material.) The behaviour shown here is compatible with the current information of Figure 5. We conclude that autophagosome puncta of micropatterned cells, which can be tracked over a longer time show strong perpendicular ordering in the periphery. However, the far greater number of puncta that can be tracked over shorter times move radially, or isotropically (Supplementary Material Figures S3–S5). A similar behaviour is observed for autophagosome puncta in non-patterned cells, suggesting a generic systems observation of perpendicular ordering.

## 4. Discussion

In this study we revealed three highly relevant phenomena concerning the dynamics and control of the autophagy system. The first finding is that a precisely enhanced autophagic flux condition with two different autophagy inducing drugs is possible. The second finding is that increased autophagic flux through rapamycin and spermidine treatment decreases autophagosome velocity and displacement. Finally, cellular and microtubule network geometry and physical constraints govern, at least in part, the autophagy response, indicated by a differential autophagosome movement and motion pattern in the central versus the peripheral intracellular region. 

### 4.1. Autophagic Flux Control is Achievable 

Although we have learnt about the autophagy machinery and its pathway regulation [25], and despite the fact that we increasingly unravel the molecular defects of autophagy in disease [2], many questions center around the tools and systems that assess the autophagic process accurately and dynamically, so that fine-tuning of autophagic activity would become possible [26]. Here, we show, to our knowledge for the first time, that the titration of enhanced autophagic flux using two drugs, rapamycin and spermidine, is possible (Figure 1). Using single cell analysis and z-stack-based pool size analysis [21], we are able to describe autophagosome flux at high resolution, allowing us to use a drug concentration that achieves a similar autophagic flux. Other commonly used techniques, such as western blotting (Figure 3), though providing valuable information about autophagy protein or cargo autophagosome abundance, are less able to resolve changes in autophagosome abundance upon bafilomycin treatment, and omit particular measurable indexes related to autophagic activity [27]. The implemented micropattern approach (Figure 2) further enhances the control over the cellular environment, allowing accurate and repeatable pool size analyses. Hence, the use of life cell imaging and single cell analysis-based fluorescence microscopy in assessing autophagic activity provides a unique and powerful tool to finely describe changes in autophagy function and dysfunction, screening libraries of autophagy modulating drugs, including spermidine and rapamycin [13,28,29,30,31,32]. Our results show enhanced autophagic activity using rapamycin and spermidine, raising autophagic flux equally, leading to an increase in respective pool size intermediates (Figure 3B,C). This is supported by others [18], who have described an increase in autolysosmal pool size upon autophagy induction. Great care must however be taken, to use saturating concentrations of a lysosomal inhibitor or deacidifying agent, such as bafilomycin [21,33], to prevent the persistence of a residual autophagic activity. Here, we utilized this scenario purposefully (Figure 1 and Figure 3), to dissect the relationship between autophagic flux and autophagy vesicle dynamics. Although this concept had been indicated recently [20,21], the direct comparison of pathway intermediates between residual and enhanced autophagic flux is of novelty (Figure 1 and Figure 3). This is important, since a dissociation between autophagy machinery function and cargo has been described [34], suggesting that, in addition to autophagic flux assessment, the particular protein cargo requires equal attention, so as not to miss dysfunction with the recognition of particular protein cargo species, but otherwise functional autophagy machinery. Although not the focus of this study, we noted that, although rapamycin and spermidine treatment equally enhanced autophagic activity based on the autophagosome pool size, the organelle size appeared larger in the rapamycin treated cells (Figure 3 and Figure S1), suggesting that cargo turnover may indeed be distinct. This deserves further attention in future studies, especially when screening drugs for efficiency and magnitude in autophagy induction, since it may highlight two major cellular control systems for tuning autophagic activity, through flux- as well as autophagosome size control. 

### 4.2. Enhanced Autophagic Flux Decreases Autophagosome Velocity 

Neurons rely on a particularly effective autophagy machinery, to maintain proteostasis. Rapamycin and spermidine have both been shown to induce autophagy in neurons, thereby enabling effective clearance of toxic protein aggregates, such as amyloid-ß and tau [14,31,32,35]. To our surprise, we show that autophagy induction using rapamycin and spermidine leads a decrease in autophagosome velocity and displacement (Figure 4A), which becomes particularly clear in the micropatterned cells. Partial inhibition of autophagosomal/lysosomal fusion however leaves velocity parameters unaffected. This is somewhat unexpected, since an increase in the turnover of the intracellular autophagosomal pool size requires an efficient autophagosomal trafficking, to prevent molecular crowding and cargo aggregation. Autophagosomes are dynein-dependently transported along the microtubulin network, which serves as linear but dynamically unstable tracks to deliver cargo for lysosomal degradation [18,36]. It is known that mutations affecting the dynein complex can lead to toxic protein aggregation. A decrease in localized ATP seems unlikely, since increased autophagy may enhance metabolite substrate generation, contributing to an energetically favorable environment [4]. In addition, also autophagosome formation is dependent on the microtubule system [37]. Since only mature autophagosomes are recruited to the microtubules [36], it is plausible that the maturation process and its control is implicated in the decrease in velocity. Whether a change in local ATP availability contributes to these findings, remains to be elucidated. Further studies will be necessary, to delineate the exact relationship between autophagic flux, ATP consumption and autophagosome transport properties. As successful aging is associated with a preserved functional protein degradation system, the molecular underpinnings that govern functional autophagosome transport for subsequent lysosomal fusion require further attention. 

### 4.3. Cell Morphology and Tubulin Network Geometry Influence Autophagosome Motion

The efficient encounter of autophagosomes with lysosomes is critical in facilitating protein degradation through autophagy. Hence, positioning of autophagy pathway intermediates as well as their pool sizes impacts on the probability for successful encounter of fusion partners. Our results highlight a differential autophagosome motion and directionality, when comparing patterned and non-patterned cells, with a clear radial motion of autophagosomes in patterned cells (Figure 5A), which, unlike in non-patterned cells, move initially towards the edge of the cell (Figure 5B). This intriguing finding highlights that cellular and microtubule network geometry and physical constraints govern, at least in part, the autophagy response. It is known that autophagosomes are formed in the cytosol, distant to lysosomes [38], and move towards the perinuclear region in a linear translocation pattern [36,39]. Micropatterning-induced microtubule organisation however, changes this pattern, strongly supporting the notion that cytoskeletal filaments, that are confined, lead to insights that a particular geometry and confinement plays an important role in vesicle dynamics [40,41]. This is of importance, given that cells forming a neuronal network of distinct susceptibility to protein aggregation [31] or a solid tumour microenvironment [42] may be characterized by inherent geometry constraints which may have an influence on the region-specific autophagic activity and response to autophagy modulation. Continued progress on the assessment of the relationship between autophagosome motion and directionality in context of lysosomal fusion will undoubtedly provide insights that unravel conditions of autophagy induction that will most profoundly favor functional protein degradation.

Taken together, it becomes clear that the precision control of autophagic activity requires the careful quantitative assessment of multiple molecular and biophysical parameters that govern functional autophagy and protein degradation. Autophagy pathway intermediate pool sizes, their positioning, their rate of fusion, motion and velocity all in the context of cellular geometry and confinement contribute to the cell’s proficiency to degrade long-lived proteins and contribute to the upkeep of proteostasis. These findings may be of importance when screening for drugs intended to fine-tune autophagic activity therapeutically, in conditions of autophagy failure in different cell and tissue types of age-associated pathologies. Hence, the utilisation of human as well as differentiated cells will be critical for future applications.

## Supplementary Materials

The following are available online at http://www.mdpi.com/2073-4409/7/8/94/s1, Figure S1: Timelapse micrographs indicating distinct pool size distribution in patterned and non-patterned cells upon autophagy induction, using rapamycin or spermidine, as well as partial inhibition, using non-saturating concentrations of bafilomycin; Figure S2: Colocalisation analysis (a) indicating colocalisation of autophagosomes with the microtubule network. Micrographs indicating colocalisation profile in patterned and non-patterned cells (b) upon autophagy induction, using rapamycin or spermidine, as well as partial inhibition, using non-saturating concentrations of bafilomycin; Figure S3: (a) Histogram for number of particles trackable up to exactly time step τ. Dataset micropatterned cells, (b) Histogram for number of particles trackable up to exactly time step τ. Dataset non-patterned cells; Figure S4: (a) Plot of $\ln\sqrt{\bar{R^{2}}}$ vs. lnτ for micropatterned cells, with the dashed line to indicate the ideal random walk scaling. Note, that the presence of such scaling is not sufficient evidence for a random walk, (b) Plot of $\ln\sqrt{\bar{R^{2}}}$ vs. lnτ for micropatterned cells, with the dashed line to indicate the ideal random walk scaling. Note, that the presence of such scaling is not sufficient evidence for a random walk; Figure S5: (a) Order measure for puncta of dataset micropatterned cells, where data are collected over tracks spanning at least τ time-steps. For tracks of longer duration strong order is noticeable in the region near the periphery. Here the maximum distance is taken as 30 units, (b) Order measure for puncta of dataset non-patterned cells, where data are collected over tracks spanning at least τ time-steps. For tracks of longer duration strong order is noticeable in the region near the periphery. Here the maximum distance is taken as 60 units.
