# Supplementary material for: The Precision Control of Autophagic Flux and Vesicle Dynamics—A Micropattern Approach
Source: Cells. 2018 Aug 3;7(8):94. doi: 10.3390/cells7080094 (PMC6116198; doi:10.3390/cells7080094)
Supplement: Supplementary file 1 [file cells-07-00094-s001.pdf]

The Precision Control of Autophagic Flux and Vesicle Dynamics—A Micropattern Approach

André du Toit [1], Sholto De Wet [1], Jan-Hendrik S. Hofmeyr [2], Kristian K. Müller-Nedebock [3] and Ben Loos [1,*]

[1] Department of Physiological Sciences, Stellenbosch University, Stellenbosch 7602, South Africa; andredt@sun.ac.za (A.d.T.); 18455468@sun.ac.za (S.D.W.)
[2] Department of Biochemistry, Stellenbosch University, Stellenbosch 7602, South Africa; jhsh@sun.ac.za
[3] Department of Physics, Stellenbosch University, Stellenbosch 7602, South Africa; kkmn@sun.ac.za

* Correspondence: bloos@sun.ac.za; Tel.: +27-218-089-196

Supplementary Materials:

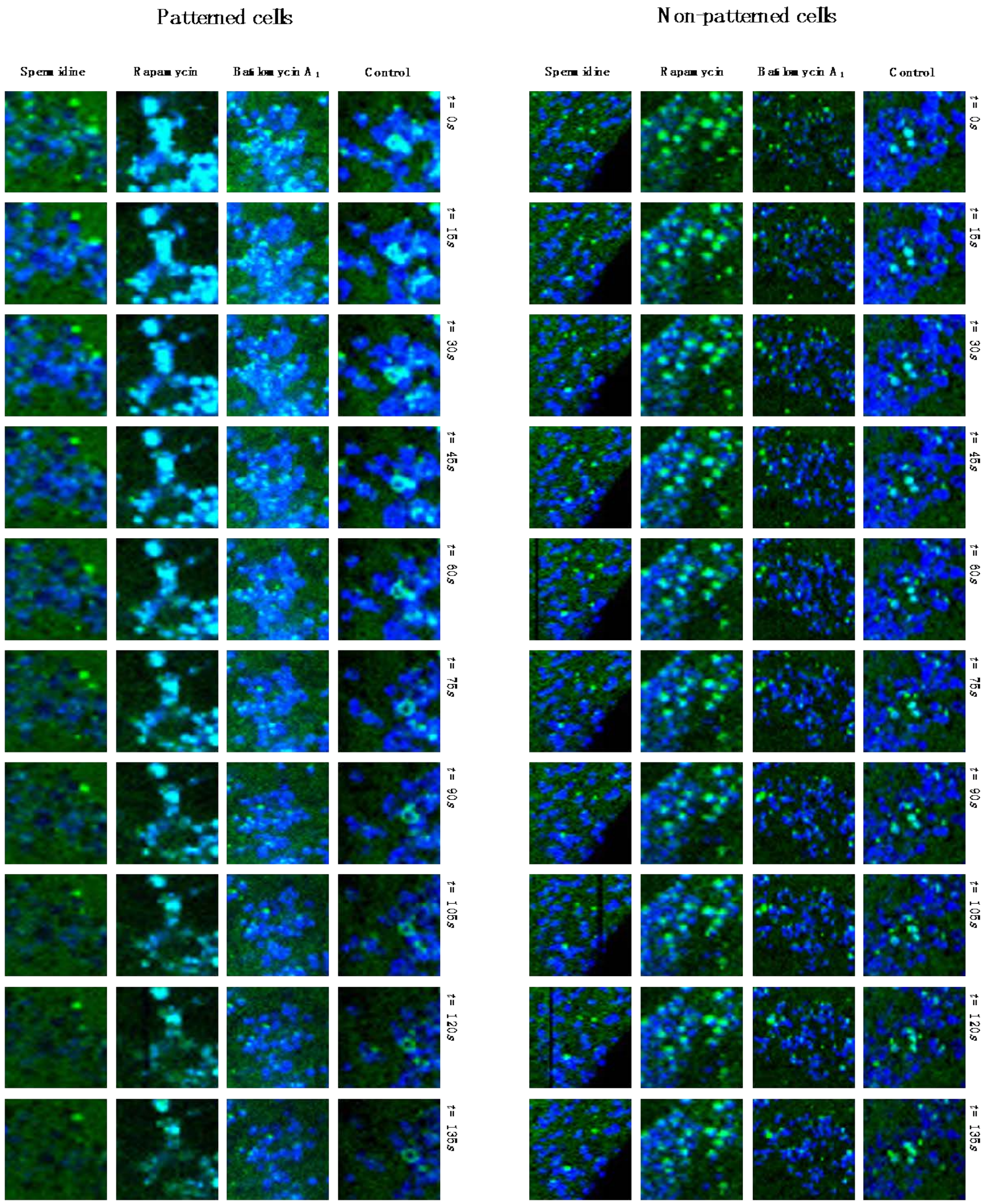

Figure S1. Time lapse micrographs indicating distinct pool size distribution in patterned and non-patterned cells upon autophagy induction, using rapamycin or spermidine, as well as partial inhibition, using non-saturating concentrations of bafilomycin.

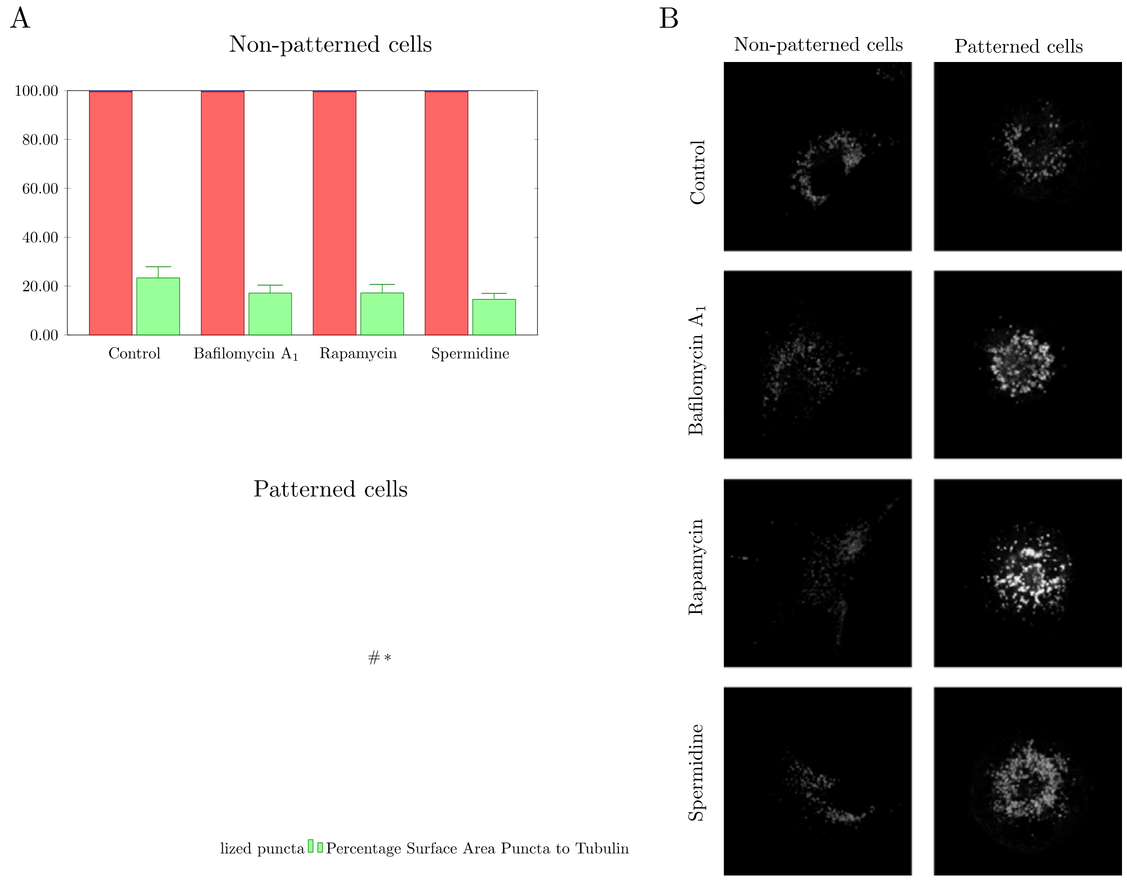

Figure S2. Colocalization analysis (a) indicating colocalization of autophagosomes with the microtubule network. Micrographs indicating colocalization profile in patterned and non-patterned cells (b) upon autophagy induction, using rapamycin or spermidine, as well as partial inhibition, using non-saturating concentrations of bafilomycin.

Data processing

Data were read into and processed using combinations of standard Mathematica® functions and graphical tools.

Tracking of particles over a number of time-steps

The figures below show the histogram of the number of puncta that can be tracked up to precisely τ time-steps from the initial time.

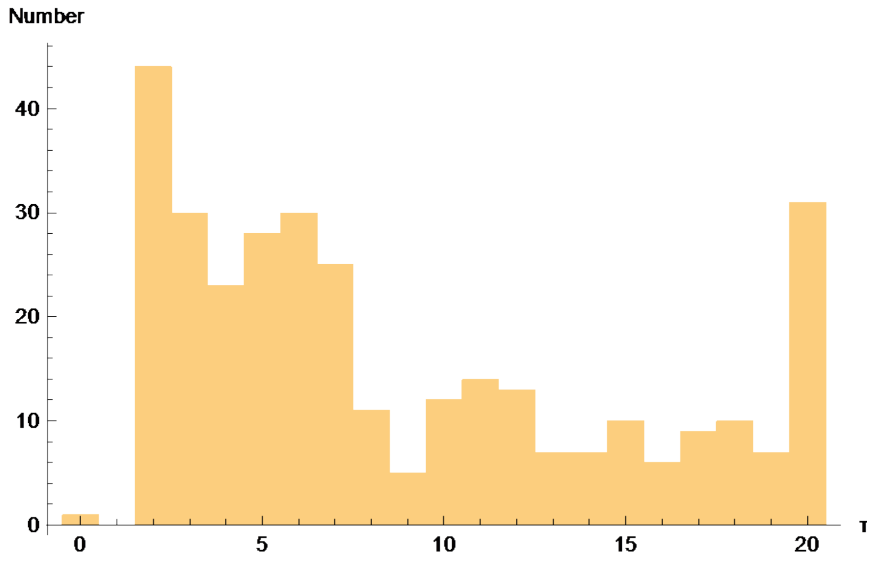

Figure S3 (a): Histogram for number of particles trackable up to exactly time step τ. Dataset micropatterned cells.

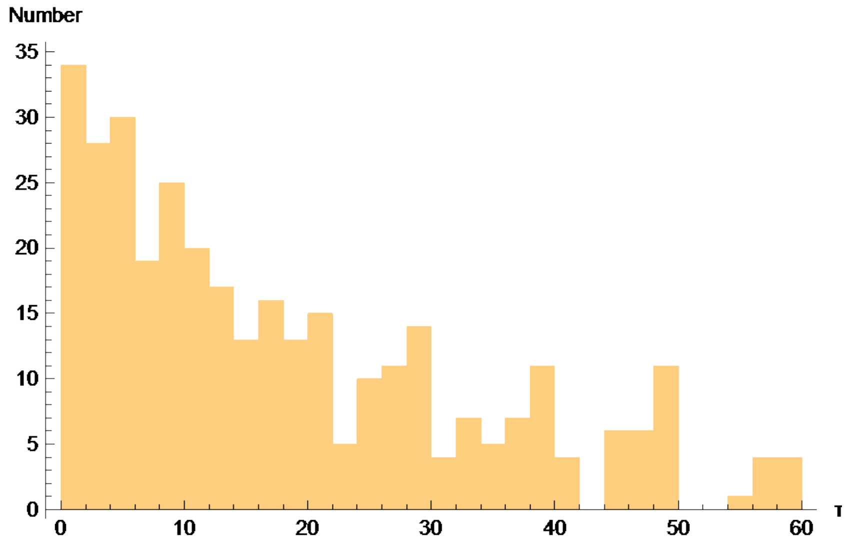

Figure S3 (b): Histogram for number of particles trackable up to exactly time step τ. Dataset non-patterned cells.

Puncta end-to-end distance distribution throughout the cells

For an ideal random walk over τ time steps, the average of the square of end-to-end distance is proportional to τ, or,

$$\overline{R^2} \sim \tau.$$

On average the puncta produce the same scaling behaviour as an ideal random walk, cf. Figure S4. However, we note that the other results of current and order with respect to a radial vector show that, in spite of a reasonable match to the ideal random walk scaling, the puncta do not behave as random walks.

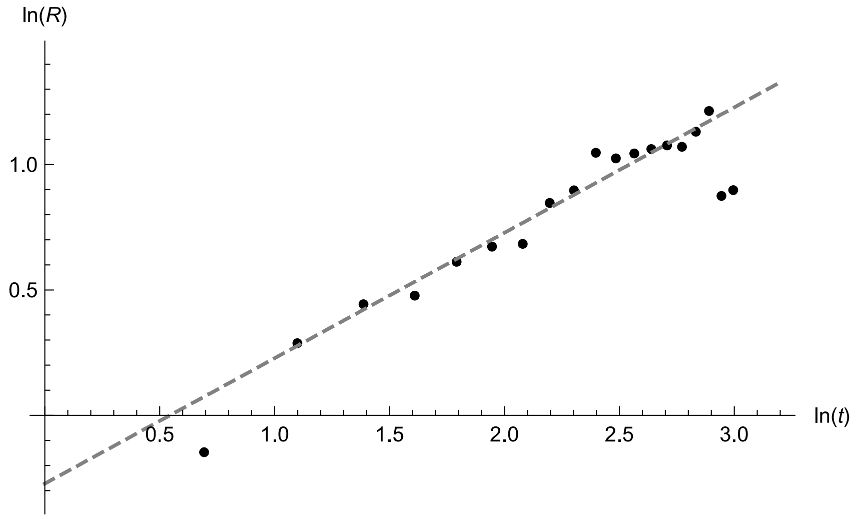

Figure S4 (a): Plot of $\ln\sqrt{\overline{R^2}}$ vs $\ln\tau$ for micropatterned cells, with the dashed line to indicate the ideal random walk scaling. Note, that the presence of such scaling is not sufficient evidence for a random walk.

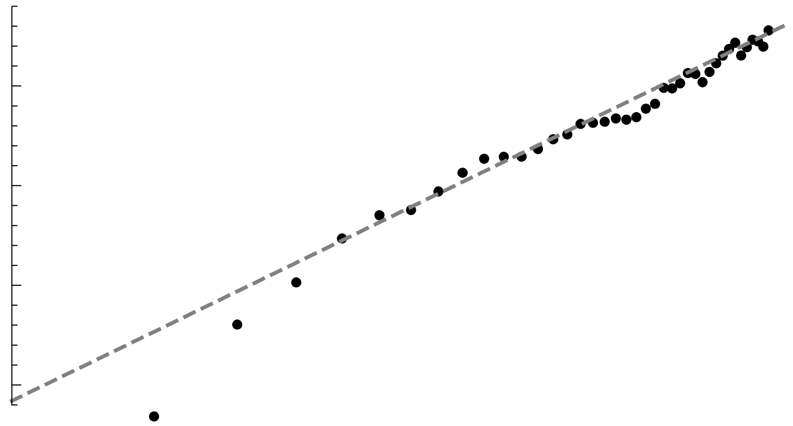

Figure S4 (b): Plot of $\ln\sqrt{\overline{R^2}}$ vs $\ln\tau$ for micropatterned cells, with the dashed line to indicate the ideal random walk scaling. Note, that the presence of such scaling is not sufficient evidence for a random walk.

Determining Q_τ

The quantity Q_τ is crucial in determining the order over more than one time-step:

$$Q_\tau = \frac{1}{N(S)} \sum_{n \in S} \left(1 - 2\left(\hat{r}_{n,\tau} \cdot \hat{v}_{n,\tau}\right)^2\right).$$

Here the quantity $\hat{r}_{n,\tau}$ is the unit vector determined from the average position of the punctum n for time-steps 1 to τ. The unit vector for the velocity $\hat{v}_{n,\tau}$ is determined by taking the difference of the

vector position of the punctum at time-step τ and time-step 1, and by diving by the number of time steps.

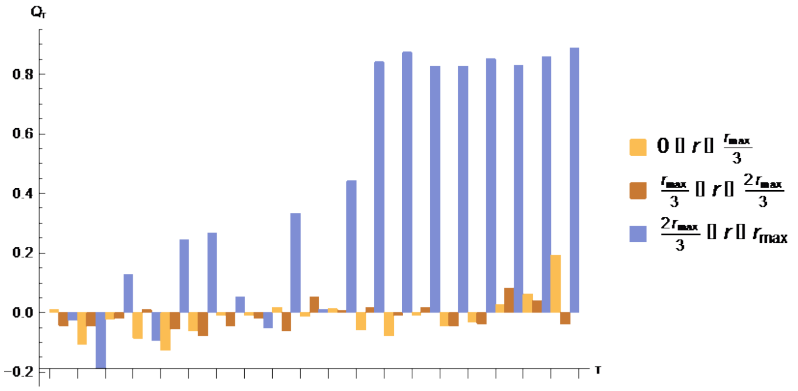

Figure S5 (a): Order measure for puncta of dataset micropatterned cells, where data are collected over tracks spanning at least τ time-steps. For tracks of longer duration strong order is noticeable in the region near the periphery. Here the maximum distance is taken as 30 units.

For non-patterned cells, we find similar behaviour of this quantity, as in the micropatterned cells described in the main text.

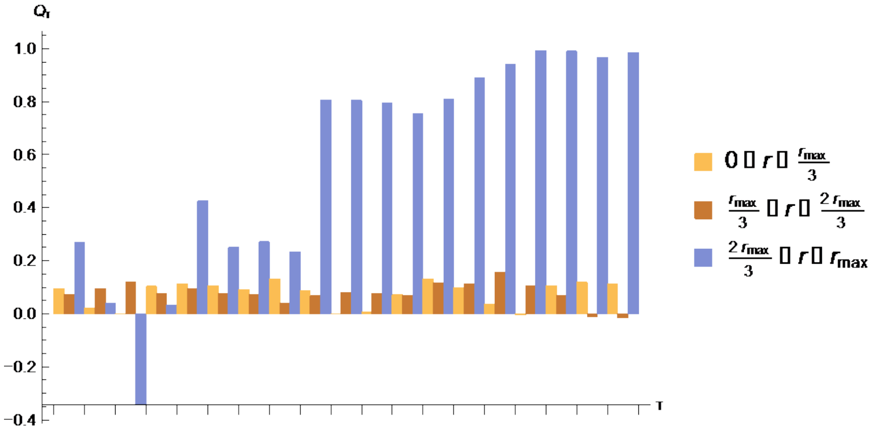

Figure S5 (b): Order measure for puncta of dataset non-patterned cells, where data are collected over tracks spanning at least τ time-steps. For tracks of longer duration strong order is noticeable in the region near the periphery. Here the maximum distance is taken as 60 units.
